# The role of receptive arts and culture to improve anxiety, depression and psychological distress in young people: a realist review

**DOI:** 10.3389/frcha.2026.1869450

**Published:** 2026-08-12

**Authors:** Daniel Hayes, Joely Wright, Louise Chandler, Kayleigh Albaya de Gago, Summi Ng, Joe Langley, Daisy Fancourt, Kamaldeep Bhui, Roisin Mooney, Rebecca Syed Sheriff

**Affiliations:** 1 Social Biobehavioural Research Group, Research Department of Behavioural Science and Health, Institute of Epidemiology & Health Care, University College London, London, United Kingdom; 2 Department of Psychiatry, University of Oxford, Oxford, United Kingdom; 3 Lab4Living, Sheffield Hallam University, Sheffield, United Kingdom; 4 Institute of Mental Health, University of Birmingham, Birmingham, United Kingdom

**Keywords:** arts, culture, mental health, realist review, receptive, young people

## Abstract

**Background:**

Arts and culture are increasingly recognised as potential supports for mental health, yet little distinction has been made between receptive engagement (e.g., visiting museums, watching performances, listening to music) and active participation (e.g., creating art or attending classes). There is limited understanding of how, for whom, and under what circumstances receptive engagement may influence anxiety, depression, and psychological distress in young people.

**Method:**

We conducted a realist review of evidence up to February 2026 to examine how receptive arts and cultural engagement may affect mental health in young people aged 16–24 years. An initial programme theory, developed through the Online Active Community Engagement programme, stakeholder consultation, and peer research, informed the search and guided analysis. We searched five academic databases and relevant grey literature. Eligible documents were screened against predefined criteria. Context–mechanism–outcome configurations (CMOCs) were developed iteratively with peer researchers and the research team to refine the programme theory.

**Results:**

Eleven documents were included. Evidence supported CMOCs indicating that receptive engagement may support mental health through: (i) short-term affective relief via attentional shift and escapism; (ii) reflection and perspective-taking through exposure to diverse viewpoints; (iii) increased belonging through perceived human connection; (iv) identity safety through representation and inclusive content; and (v) support for emotion regulation when engagement is regular and accessible. Effects were most consistent for proximal outcomes during or shortly after engagement, with limited evidence for sustained reductions in anxiety, depression, or psychological distress beyond the engagement period.

**Conclusions:**

Receptive arts engagement may provide an acceptable, low-stigma form of support that complements, rather than replaces, clinical care. Benefits may occur across the continuum of need and contribute to prevention, particularly when engagement is relevant, inclusive, and sustained. Evidence for longer-term symptom reduction remains limited and warrants further investigation.

**Systematic Review Registration:**

https://www.crd.york.ac.uk/PROSPERO/view/CRD42023476421, PROSPERO 275 (CRD42023476421).

## 1 Introduction

The last 20 years have seen rapid interest in, and research on, the impact of arts and culture in improving health (1). In the UK, an inquiry report from the UK All-Party Parliamentary Group on Arts, Health and Wellbeing illustrated the significant impact on personal and public health that engagement in activities across a spectrum, from clinical arts interventions to non-clinical, community-based arts and cultural programmes, can have (2). Globally, other countries are also developing policy and programmes around arts and health (3), and bodies such as the World Health Organization have increasingly promoted the role of arts and cultural engagement across prevention, health promotion, and the management of illness (1).

Whilst broad in scope, engagement with arts and culture encompasses a wide range of activities, including performative arts (e.g., singing, dancing), visual arts and craft (e.g., drawing, making), literature and storytelling, and cultural participation (e.g., visiting museums and galleries, in person or virtually) (4). These activities can also be distinguished as active or participatory versus receptive depending on the degree of active involvement required (e.g., creating or performing versus attending, observing, listening, reading or consuming) (1). Receptive engagement may be particularly important to understand because it can include both attendance-based cultural experiences and everyday self-directed forms of engagement, such as listening to music, engaging with digital arts content, reading literature or viewing performances online (1, 5). This may make it comparatively lower-intensity, scalable and, in some forms, more autonomously accessible than structured participatory programmes. Receptive engagement may also involve distinct mechanisms, including aesthetic absorption, perspective-taking, symbolic meaning-making and identification with narratives or representations, which may have particular relevance for mental health and wellbeing (6, 7).

The role of arts and culture fits with the principles of mental health recovery and person-centred care, which have increasingly shaped contemporary mental health services in many Western countries (8, 9). Specifically, these approaches emphasise emotional, social and existential needs alongside clinical care, aligning with arts and cultural activities as non-clinical resources that may support wellbeing, meaning, identity, connection and hope (10). In some settings this has led to more structured models, such as Arts on Prescription, whereby patients are offered or referred to arts and cultural activities through pathways linked to healthcare or community support. Although previous reviews of Arts on Prescription have highlighted methodological limitations, including small sample sizes and limited use of control groups, the overall evidence base suggests promise for outcomes relevant to anxiety and depression (11, 12). A recent controlled trial in Greece among adults receiving Arts on Prescription alongside standard treatment reported reductions in anxiety and depression and improvements in wellbeing compared with standard treatment alone, and included receptive activities such as theatre, cinema, museum visits and music events (13). These examples suggest receptive engagement may operate both within formal care pathways and outside them as part of everyday life.

Receptive arts and cultural engagement may be most relevant in supporting mental health and wellbeing as a preventative approach to mental health maintenance, and self-management, potentially reducing the severity, duration, or escalation of distress and delaying or preventing progression to formal service use. Epidemiological evidence across the lifespan suggests positive associations between arts and cultural engagement and mental health outcomes (14–16). However, despite a growing evidence base, much of the research is situated in adult populations. This may reflect a combination of the typical time lag between adult evidence and translation to youth settings, as well as historic underinvestment in youth mental health relative to adult mental health in many contexts (17).

This focus on young people is particularly important given the high and rising prevalence of emotional difficulties and psychological distress (18). In England, around one in five children and young people aged 8–25 met the threshold for a probable mental disorder in 2023 (19). Beyond substantial impacts on quality of life and functioning, there are large societal and economic costs associated with youth mental health difficulties, including effects on education, employment trajectories, and productivity across the life course (20). In particular, anxiety and depressive disorders are among the most prevalent and burdensome mental health conditions in adolescence and young adulthood, contributing substantially to disability and reduced functioning during a key developmental period (21). Psychological distress is an important complementary outcome because it captures broader, non-specific mental health strain, including subthreshold and mixed presentations, that may not meet diagnostic thresholds yet still confer impairment and elevated risk for later disorder and service need (22).

While evidence for arts and culture in supporting mental health is growing, much of the evaluative literature has focused on structured, facilitated or participatory forms of engagement, such as arts programmes and group-based activities (1). In contrast, receptive engagement has received comparatively less attention as a distinct exposure, despite potential relevance for everyday coping, prevention and early self-management (1, 6). Receptive activities may be particularly relevant for subthreshold distress, where lower-intensity and self-directed supports may be appropriate to prevent escalation and the need for formal help seeking. They may also be more feasible for young people experiencing low mood, anxiety, fatigue, withdrawal or low confidence, and easier to integrate into routine life than participatory programmes requiring sustained energy, social exposure or skills-based participation. Importantly, receptive engagement may afford young people greater agency and control over whether, when and how they engage, which may be especially relevant given well-established barriers to help-seeking and concerns about loss of autonomy within formal support pathways, particularly for young people from underrepresented backgrounds (23, 24).

Receptive engagement may also have distinct implications for equity. Access to cultural opportunities can be shaped by background, geography, socioeconomic position, disability, cost, transport, and whether cultural spaces feel inclusive, safe, relevant, and offer opportunities for recognition or feeling “seen,” (25, 26). Digital and online cultural engagement may mitigate some access barriers by enabling low-cost or remote participation but may also reproduce or create inequities linked to differential access to technology and the ways content is curated and distributed (27, 28). Understanding how contextual factors shape engagement and benefit is therefore critical for designing approaches that are not only effective, but equitable and implementable.

Engaging in the arts, whether through receptive or participatory means, may contain “active ingredients” that are beneficial to mental health, including cognitive stimulation, social interaction, multi-sensory engagement, and opportunities for creativity, imagination, and meaning-making (29). Mechanistically, these ingredients may activate psychological, biological, social and behavioural pathways, including supporting emotion regulation, reducing stress responses, shifting maladaptive cognitions, fulfilling psychological needs (e.g., autonomy, competence, and relatedness), supporting identity development and personal growth, and strengthening social connection and support networks (7). However, effects are unlikely to be uniform: outcomes may plausibly vary according to the context in which engagement occurs, the accessibility and acceptability of activities, and whether engagement becomes a sustained routine or occurs during specific periods of distress or transition.

To our knowledge, no realist synthesis has examined receptive arts and cultural engagement as a distinct exposure for youth anxiety, depression and psychological distress, nor explained the contexts and mechanisms through which effects may arise. We therefore conducted a realist review to develop and refine a programme theory explaining how receptive arts and cultural activities might support mental health outcomes in young people, and to identify the key contexts and mechanisms through which effects may occur.

## 2 Materials and methods

The review protocol was registered on PROSPERO (CRD42023476421).

Realist reviews are often used in the context of interventions affecting health as a way of explaining how interventions work, for whom, in what circumstances and why (30). They are underpinned by a generative causation stance; contexts may trigger specific mechanisms, which can lead to outcomes. It involves the development of context-mechanism-outcome configurations (CMOCs). These CMOCs inform and are brought together within a programme theory—an explanation of how an intervention works, for whom, under what conditions and why (30).

### 2.1 Study selection

Data sources were identified through formal searches using both academic and grey literature. This included searching the following (i) academic databases: MEDLINE, Embase, PsycINFO, the Cochrane Library, and the Web of Science which took place from database inception up to 28th February 2026. (ii) websites and data archives detailed in Supplementary Material (S1). (iii) reference searching for all included papers, and (iv) asking relevant stakeholders and subject experts to suggest any papers or reports they deemed relevant.

### 2.2 Inclusion criteria

To be eligible, documents were required to meet criteria relating to participants, interventions/exposure, comparators and outcomes (PICO). We included documents involving young people aged 16–24 years, selected to reflect common international definitions of youth/young people, to capture a transitional developmental period during which onset of anxiety and depression commonly peaks, and to focus on an age range in which autonomy and independent engagement with arts and cultural activities may be particularly salient (31–33). Participants were required to have anxiety and/or depression, identified through self-report, clinician report, or validated symptom measures, or elevated psychological distress consistent with probable anxiety and/or depression.

We included receptive arts and cultural engagement, defined as attendance, observation or consumption of arts and culture (e.g., visiting museums or galleries; watching theatre, dance or film; listening to music; engaging with online arts and cultural content), including engagement delivered digitally. Digital arts and cultural content referred to artistic, cultural or heritage material accessed through digital technologies, such as virtual museums and galleries, online exhibitions, recorded performances, digitally delivered narrative content, and curated cultural collections, where engagement was primarily observational rather than participatory. Activities involving active artistic creation or participatory arts as the primary mode of engagement were excluded. Where documents included hybrid or boundary-spanning cultural forms (e.g., narrative practices, immersive cultural environments, or cultural community participation), these were retained when they provided mechanism-rich data that could inform refinement of the programme theory, but were interpreted as explanatory analogues rather than direct evidence of receptive engagement effects. We included all study types (quantitative, qualitative, mixed methods, case studies and evaluations) to support realist theory development. No language restrictions were applied during searching. However, non-English documents were excluded at full-text screening where no member of the research team or peer researchers could reliably interpret the content.

### 2.3 Search strategy

A search strategy was developed to map onto the PICO criteria. Youth populations were captured using terms such as “youth*”, “student*”. Intervention terms were included to capture the range of possible programmes targeting the arts and culture, such as “art”*, “museum*”. Finally, to identify studies with relevant outcomes, mental health terms such as “depress*” and “distress*” were included. For the search strategy, see Supplementary Material (S2).

### 2.4 Youth involvement and reflexive input

Two peer researchers aged 16–24 years (both female), including one describing themself as White British and the other describing themselves as from Hong Kong. Their involvement extended beyond advisory roles and included informing search terminology, interpreting evidence, challenging researcher assumptions, and contributing to the iterative refinement of context-mechanism-outcome configurations (CMOCs) and programme theory. This involvement strengthened the relevance and reflexivity of the review by incorporating perspectives from young people with direct experience of mental health difficulties and arts and cultural engagement.

### 2.5 Screening and selection

**Figure 1:**
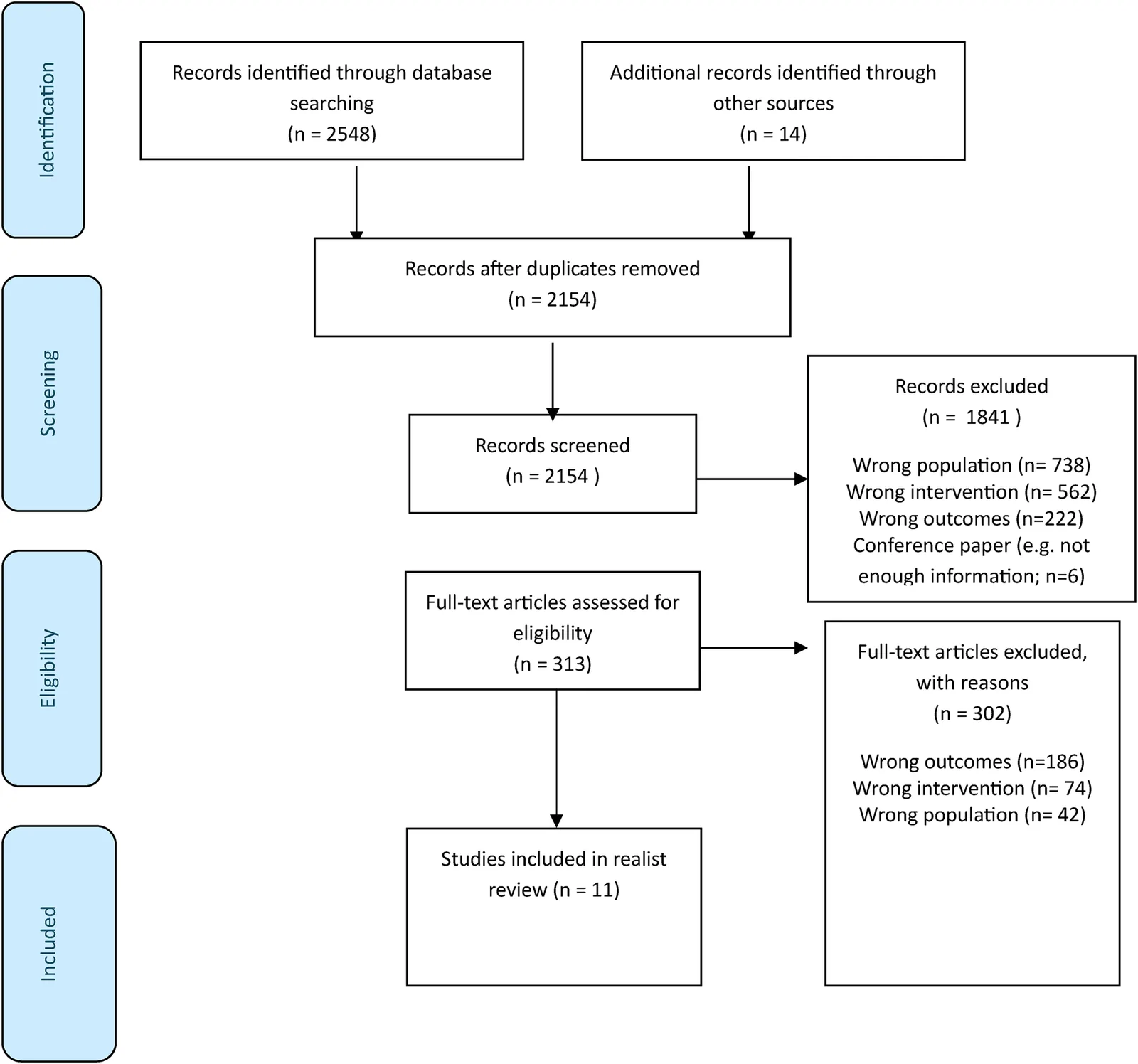
PRISMA 2020 flow diagram of study identification and selection. Adapted from Page et al. (2021) under the Creative Commons Attribution 4.0 International licence (CC BY 4.0). The template was modified and populated with data from the present review.

Search results were exported to Excel and duplicates removed. Screening took place in two stages. First, two authors (DH and JW) independently screened titles and abstracts against eligibility criteria, resolving disagreements through discussion. Second, full-text screening was conducted by the same two authors for all remaining documents, with uncertainties resolved by discussion and, where needed, adjudication by a reference group (RS and RM) to reach consensus. Although the protocol initially anticipated screening by three reviewers, screening was undertaken by two independent reviewers throughout, with disagreements resolved through discussion and, where required, adjudication by the reference group. The flow of records from searching through to inclusion is summarised in Figure 1.

### 2.6 Data analysis

Articles were imported into NVivo 14 (34) for data extraction. A realist logic of analysis was employed in analysing the data from included documents. Data extraction began using sensitising concepts informed partly by the Online Active Community Engagement (O-ACE) programme theory (see Supplementary Material S3), while remaining open to mechanisms that extended, contradicted or re-specified the initial theory and other topics identified in the included papers. Interpretive cross-case comparison and retroductive reasoning aimed to establish explanations of how and why the context had influenced observed outcomes and the mechanisms by which this took place (35). Exploring the data in this way enables evidence-based hypotheses to gradually accumulate and for these to be drawn together into CMO configurations. Using substantive theory as a broad conceptual framework during realist research can provide a structure within which to organise the numerous programme theories arising from the data (36). Data from coding was completed in four stages over 6 months, led by DH and supported by another researcher (JW). The previously developed programme theory was initially used to develop broad themes (e.g., “how the intervention is delivered” and “intervention medium”) to begin to think about CMOCs. After this stage, CMOCs and thinking were presented back to peer researchers and the wider research team for critical appraisal, reflexive challenge, and further reflection. The researchers then re-examined the data again, refined CMOCs and repeated this process.

### 2.7 Quality reporting

The RAMESES (Realist And Meta-narrative Evidence Syntheses: Evolving Standards) reporting guidelines for realist reviews were followed by the research team (37). This involved the researchers considering whether included articles contained useful data for extending or testing the emerging CMOCs and/or programme theory (i.e., “relevance”) and examining whether the piece of data used was underpinned by credible and trustworthy methods (i.e., “rigour”). RAMESES reporting is outlined in Supplementary Material (S4). In practice, documents judged to provide higher rigour and higher relevance (mechanism-rich data) were weighted more heavily in CMOC development, while lower-rigour evidence was used cautiously to suggest or triangulate candidate mechanisms rather than to support strong claims.

## 3 Results

Database searching yielded 2,154 records, while expert consultation and reference searching yielded an additional 6 and 8 records respectively. Following title and abstract screening, 1,841 records were excluded. The most common reason for exclusion at this stage was the wrong population (*n* = 738). Full-text screening excluded 302 documents, resulting in 11 included documents (Figure 1). The most common reason for exclusion at full text screening was wrong outcomes (*n* = 186). Included documents consisted of qualitative, survey, quasi-experimental and randomised designs, with most evidence generated during or shortly after the COVID-19 pandemic (2020–2022). Study characteristics (including design, population, setting, type of receptive engagement, and outcomes assessed) are summarised in Table 1.

**Table 1:** Characteristics of included studies and contribution to programme theory.

Study	Country/setting	Population and age	Design	Receptive engagement exposure	Comparator	Outcomes/constructs assessed	Contribution to programme theory
Bottorff (38)	Conceptual, transformational festival contexts	Young adults (conceptual focus), not specified	Interpretive/conceptual analysis	Immersive cultural participation	None	Meaning-making, catharsis, affective release	Conceptual elaboration of symbolic and affective mechanisms
Chen et al, (39)	Taiwan, university setting	Nursing students with depressed mood (aged 16–20)	Randomised trial	Structured music listening	Control condition	Depressive symptoms, cortisol	Evidence supporting repeated receptive engagement and symptom change
Chmiel et al. (40)	Australia, COVID-19 lockdown context	Adults including young adult subgroup (18–24)	Cross-sectional survey	Creative and receptive arts engagement, particularly music	None	Anxiety, depression, perceived helpfulness	Contextual evidence on content preferences, escapism and emotion functions
Goodman & Newman (41)	USA, school setting	Adolescent girls (aged 14–18)	Quasi-experimental	Oral and digital storytelling	Modality comparison	Stress, anxiety, anger, depression	Mechanism-rich evidence on validation, narrative processing and developmental variation
Hides et al. (42)	Australia, digital intervention	Distressed young people (aged 16–25)	Randomised controlled trial	Music listening via Music eScape app	Delayed-access control	Emotion regulation, distress, wellbeing	Evidence on repeated engagement and boundary conditions linked to unhealthy music use
Reid (56)	USA, school and community cultural setting	Black LGBTQ + young people (aged 16–19)	Qualitative study	Identity-affirming cultural spaces (ballroom culture)	School context comparison	Belonging, identity safety, wellbeing	Evidence supporting identity safety and belonging mechanisms
Syed Sheriff et al. (43, 44)	UK, online	Young people (aged 16–24)	Linked protocol and co-design study reports	Online arts and cultural engagement (Ways of Being)	Planned comparator	Planned affective and mental health outcomes; acceptability	Initial programme theory and mechanism hypotheses
Syed Sheriff et al. (45)	UK, online	Young people and wider online sample	Cross-sectional survey	Online cultural content	None	Perceived mental health and wellbeing benefits	Evidence supporting routinisation and frequency as enabling conditions
Syed Sheriff et al. (46)	UK, online	Young people (aged 18–24)	Qualitative interview study	Online arts and culture	None	Perceived mechanisms and outcomes	Evidence for human connection, reflection and attentional shift
Syed Sheriff et al. (47)	UK, online	Young people (aged 16–24)	Parallel-group RCT	Co-produced online cultural experience	Typical museum website	Negative affect, positive affect, distress	Experimental evidence for proximal affective relief
Zhou et al. (48)	China, museum setting	Young visitors (aged 15–24)	Pre–post survey study	Art museum visits	Pre–post comparison	Positive and negative affect	Evidence extending proximal affective effects beyond online contexts

**Figure 2:**
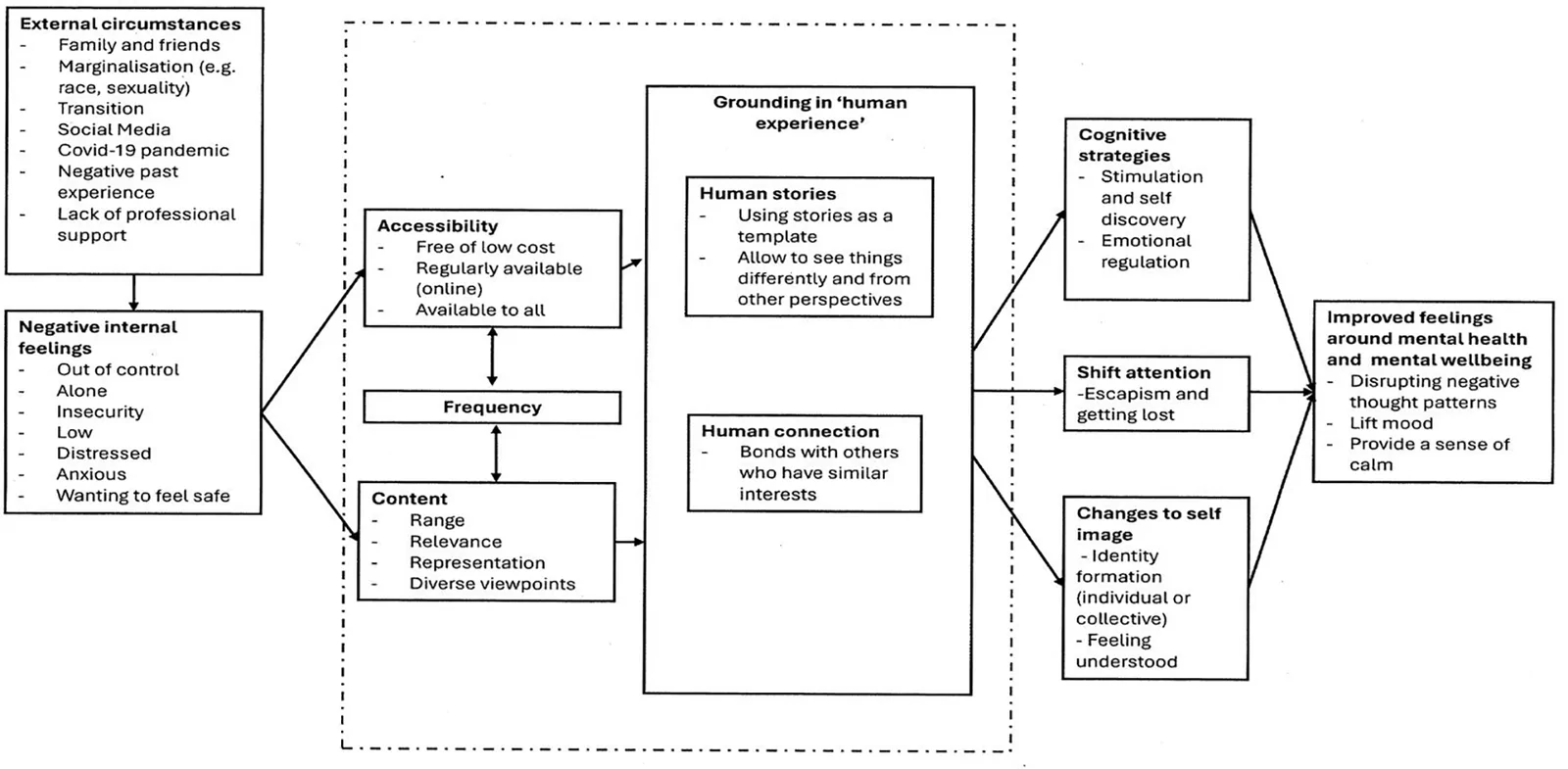
Programme theory.

Findings are organised to reflect iterative refinement of the programme theory, progressing from contextual conditions and delivery features through candidate mechanisms and their proximal and conditional distal outcomes. Realist analysis led to the development of 18 CMOCs (Supplementary Material S5). These CMOCs were iteratively discussed with peer researchers and the wider team and were used to refine the initial programme theory (Supplementary Material S6). Further iteration with researchers and peer researchers led to the development of the final programme theory in Figure 2.

The refined programme theory highlights how receptive engagement with arts and culture may contribute to youth mental health outcomes through interacting domains of (i) delivery and accessibility, (ii) frequency and routinisation, (iii) content features (including relevance, representation and viewpoint diversity), and (iv) mechanisms linked to attention shift, connection and emotional processing.

Although the focus of this review was receptive engagement, several included documents examined hybrid or boundary-spanning cultural forms that combined receptive and participatory elements. These studies were retained because they provided theoretically informative evidence regarding mechanisms such as belonging, identity safety, emotional processing, meaning-making, and human connection. Consistent with realist methodology, these studies were used primarily to refine and elaborate programme theory rather than as direct evidence of the effectiveness of receptive engagement itself.

Four studies from the O-ACE programme generated evidence across five linked papers examining online arts and culture (OAC) for young people (43, 44), a proof-of-principle parallel-group RCT (46), and additional survey and interview studies (45, 47). Although several arose from a related programme of work, they contributed distinct forms of evidence (co-design, trial, survey and qualitative data) and were treated as linked but analytically distinct contributions.

Beyond OAC, an RCT of a music-based emotion regulation app in psychologically distressed young people reported good acceptability but no short-term between-group differences at one month, with improvements over time and moderation by baseline “unhealthy music use” (for example, using music in ways that reinforce rumination or negative memories) (42). A quasi-experimental evaluation comparing oral and digital storytelling in adolescent girls examined changes in stress, anxiety and depression outcomes, with variation by modality and grade level (41). Survey evidence during lockdowns documented high engagement in creative activities and suggested young adults particularly favoured musical engagement, with patterns consistent with emotion and identity functions (40). Finally, qualitative work on identity-affirming cultural spaces (49) and conceptual analysis of immersive “transformational festival” contexts (38) broadened the explanatory base for mechanisms linked to belonging, identity safety, meaning-making and catharsis. A further survey-based pre–post study of 306 young museum visitors in China reported increases in positive affect and reductions in negative affect following art museum visits, extending evidence for receptive cultural engagement beyond online settings (48).

Across the quantitative studies, outcomes included affect, psychological distress, and symptoms of anxiety and depression. The strongest controlled experimental evidence for short-term change came from the Ways of Being trial, which reported reduced negative affect over the intervention phase but no sustained differences at six weeks and no group differences in positive affect or psychological distress (46), when comparing online museums, one of which was based on human stories. Goodman and Newman (2014) reported changes in stress, anxiety and depression following storytelling, with effects varying by modality and grade (41). By contrast, the Music eScape RCT did not detect short-term between-group differences at one month, despite improvements over time in both groups (42).

### 3.1 External circumstances and impact on feelings

Across studies, these external circumstances functioned as contextual constraints and affordances that shaped the availability, salience and perceived value of receptive engagement, rather than operating as mechanisms in themselves. Findings supported a context-dependent configuration in which receptive arts and cultural engagement was most commonly described as a coping response during periods of stress, isolation, or constrained access to support. Survey evidence during COVID-19 restrictions reported that OAC engagement was perceived as helpful for mental health and wellbeing, with benefits concentrated among regular users (45). The O-ACE programme papers similarly situate online cultural experiences within a context of unmet youth mental health need and barriers to formal help-seeking, proposing cultural engagement as a scalable and acceptable alternative route to support (43).

Beyond pandemic-related constraints, external context also included identity-related stressors and exclusion. Reid (2022) documented how school contexts shaped isolation and anxiety/depression risk for Black LGBTQ + young people through fear of rejection and pressure to suppress identity, while engagement with ballroom culture (i.e., watching dance battles in the queer community) functioned as a safer, affirming environment supporting confidence and self-expression (49). Bottorff's (2015) analysis of immersive festival environments, included as a theoretically informative hybrid cultural form, further conceptualises immersive cultural participation as a response to loneliness and meaninglessness. The Bottorff study involved an immersive cultural environment that combined receptive and participatory elements. Although participants could actively contribute to the festival environment, the mechanisms informing the present programme theory primarily related to observation, exposure to artistic and cultural artefacts, and encounters with narratives and shared symbolism. We therefore interpreted this study as an explanatory analogue rather than direct evidence of receptive engagement, using it to refine mechanisms rather than infer effectiveness, proposing that cultural events may support emotional release and meaning making (38). Together, these documents indicate that receptive arts and culture may be sought particularly when young people experience psychological strain or limited access to acceptable support options.

### 3.2 Delivery: accessibility (including online) and acceptability

Evidence was consistent with delivery-related configurations in which feasibility and acceptability enable engagement. Chmiel et al. (2022) reported widespread engagement in artistic creative activities during lockdown and identified music listening as particularly salient for younger adults, suggesting that accessible and everyday cultural practices are commonly used to support feeling better (40). In the Music eScape RCT, digital delivery supported accessibility, but engagement varied and some participants reported technical barriers, indicating that usability and implementation factors can constrain uptake even when interventions are available (42)^.^

The most direct evidence of online cultural delivery producing measurable benefit comes from the Ways of Being RCT. Compared with a typical museum website, the co-produced cultural experience reduced negative affect over the intervention phase, suggesting that online provision can support short-term affective relief (46). The stories based intervention (Ways of Being) was limited to only 11 stories and effects were not sustained at six weeks and were not observed for psychological distress. Complementing online evidence, a pre–post museum study suggested in-person receptive engagement may also support short-term affective relief, with post-visit increases in positive affect and reductions in negative affect among young museum visitors (48). This may suggest that broader content, or sustained engagement over time, could be important for longer-term outcomes.

Acceptability and non-stigmatising framing also emerged as important delivery conditions. The O-ACE programme explicitly frames online culture as a non-clinical route to supporting mental health, potentially lowering barriers for young people reluctant to seek clinical help (43). Co-design work from the O-ACE programme further suggested acceptability may have been strengthened through youth involvement in shaping content, language and navigation, supporting fit with young people's preferences and needs (44). Participant feedback from the Ways of Being trial reinforced this acceptability pathway, highlighting the value of the experience not being “therapy” (46). While accessibility and low barriers to entry supported uptake and acceptability, they were not sufficient on their own to produce deeper or sustained outcomes without additional enabling conditions.

### 3.3 Frequency

Across the evidence base, benefits were more consistently reported where engagement was regular rather than occasional. Syed Sheriff et al. (2022) found that almost all perceived benefits of OAC were associated with regular use (defined as at least monthly engagement) (45). Qualitative interviews similarly suggested that familiarity and routine engagement strengthened perceived effects, including improved calmness, lifted mood and disruption of negative thought patterns (46). Evidence from the Music eScape trial suggests that changes may require time or repeated practice: although no between-group differences were observed at one month, improvements were detected over longer follow-up, which is consistent with the possibility that repeated engagement may support skill development and wellbeing (42). Additional support comes from a music-listening intervention delivered twice weekly over 10 weeks, where reductions in depressive symptoms were observed, consistent with the possibility that repeated receptive engagement may support cumulative benefits (39). These findings support the O-ACE programme theory that regular receptive engagement can provide a consistent avenue for managing feelings and maintaining wellbeing. Taken together, these findings indicate that frequency and routinisation operate as key enabling conditions, shaping whether receptive engagement remains a short-term coping response or contributes to more sustained emotional regulation, rather than operating as mechanisms in themselves.

### 3.4 Content: range, relevance, representation and diverse viewpoints

Content emerged as a key mechanism-bearing dimension across studies. Survey evidence supports the value of offering a range of activities that align with youth preferences: Chmiel et al. (2022) found younger adults particularly favoured musical engagement, underscoring the importance of aligning options with user preferences (40). The Ways of Being intervention design similarly prioritised exploration through interconnected stories and viewpoints, increasing the likelihood that young people would encounter content that felt relevant and engaging (46).

Relevance of content to emotional state and lived experience was particularly salient in the storytelling evaluation. Goodman and Newman (2014) illustrated how digital storytelling enabled adolescents to express difficult experiences and be understood by others, with the vignette (“I get it now”) suggesting validation as a plausible mechanism (41). The study also reported changes across stress, anxiety and depression outcomes, with variation by modality and grade, implying that developmental stage may shape which forms of narrative engagement are most effective. Music-based approaches provide complementary evidence that emotion-relevant content may support coping, while also requiring attention to how engagement is enacted. Hides et al. (2019) found moderation by baseline “unhealthy music use”, such as engaging with music in ways that reinforce rumination or negative memories, indicating that benefits may depend partly on how music is used for emotion regulation, including whether engagement reflects rumination or supports more adaptive regulatory processes (42). Complementing this, culturally structured music listening has also been associated with reductions in depressed mood, suggesting emotional and cultural qualities of content may shape therapeutic potential (39).

Representation and inclusivity were prominent in understanding who benefits. Reid (2022) demonstrates how identity-threatening contexts contribute to isolation and increased depression/anxiety risk, while identity-affirming cultural spaces support safety, expression and belonging (49). In the Ways of Being trial, subgroup patterns were reported in which the intervention showed stronger negative affect reductions for groups such as ethnic minority participants and males, alongside feedback emphasising inclusivity and reduced feelings of being alone (46). Together, this supports the ORIGIN proposition that content representation may moderate engagement and benefit for underrepresented young people, by activating mechanisms of identity safety, validation and belonging, rather than representation constituting an outcome in itself.

Diverse viewpoints and human stories were repeatedly linked to reflection and perspective. Qualitative OAC accounts described how exposure to other experiences supported learning, reflection and mood improvement (46, 47). This is consistent with the O-ACE theory of change, which prioritises human-centred narratives as a mechanism-bearing feature of the intervention (43, 44). Bottorff's (2015) conceptual analysis provides a broader parallel, proposing that cultural immersion may promote meaning-making and emotional release through narrative, symbol and collective experience (38). Across studies, representation and perceived relevance consistently functioned as mechanism-enabling conditions, supporting identity safety and reducing the emotional costs of concealment or marginalisation.

### 3.5 Grounding in human experience, human connection, and shift in attention

Mechanisms identified across studies appeared to operate across different timescales, with consistent evidence for proximal affective relief and more conditional support for mechanisms linked to reflection, meaning making, and emotion regulation.

Mechanisms grounded in human experience—including connection, belonging and attentional relief—were among the most consistently supported across study types. Story-based engagement supported emotional processing and feeling understood (41) while OAC qualitative evidence highlighted human connection as a key perceived mechanism for mental health benefit (46). Identity-affirming cultural community participation in Reid (2022) further demonstrates how cultural belonging may counteract isolation and support wellbeing (49).

Attentional shift was a recurrent explanatory pathway for short-term relief. Chmiel et al. (2022) synthesised evidence that arts engagement supported wellbeing through escapism and avoidance-based emotion regulation (40), and the Ways of Being trial provides partial experimental support for proximal affective relief through reduced negative affect (46). However, Hides et al. (2019) indicate a potential boundary condition: baseline patterns of unhealthy music use moderated changes, suggesting that attentional escape may not always reduce distress if engagement reinforces negative mood states.

### 3.6 Outcomes

Across the included studies, outcomes associated with receptive arts and cultural engagement clustered into three broad levels within the refined programme theory (Table 2): proximal outcomes, intermediate psychological and social outcomes, and distal mental health outcomes. Proximal outcomes were the most consistently observed and included short-term reductions in negative affect, perceived stress, and emotional relief following engagement. Intermediate outcomes comprised enhanced emotion regulation, reflection, perspective-taking, meaning-making, identity safety, feeling understood, belonging, and social connection. Distal outcomes referred to broader improvements in symptoms of anxiety, depression and psychological distress. Overall, evidence was strongest for proximal outcomes, whereas evidence for distal mental health improvements was more variable and appeared contingent on repeated engagement and supportive contextual conditions.

**Table 2:** Outcome hierarchy within the refined programme theory.

Outcome level	Examples identified in the review
Proximal outcomes	Reduced negative affect, improved mood, calmness, stress relief, attentional shift, temporary psychological distance from stressors
Intermediate psychological and social outcomes	Improved emotion regulation, coping confidence, reflection, perspective-taking, meaning-making, identity safety, feeling understood, belonging, social connection
Distal outcomes	Reduced symptoms of anxiety, reduced symptoms of depression, reduced psychological distress, improved longer-term mental health

Proximal outcomes were consistently reported across qualitative, survey and experimental studies and included improvements in mood, reductions in perceived stress, and short-term emotional relief following engagement. Evidence for more sustained improvements in anxiety, depression and psychological distress was less consistent and was primarily reported in studies involving repeated or more sustained engagement. Across the evidence base, intermediate outcomes such as emotion regulation, belonging, reflection and identity safety appeared to provide plausible explanatory pathways linking immediate affective changes to longer-term mental health outcomes, informing the refined programme theory presented below.

### 3.7 Refined programme theory

Drawing together these findings, the refined programme theory (Figure 2) proposes that receptive arts and cultural engagement may support youth mental health through interacting mechanisms operating across delivery, frequency and content. Several mechanisms, particularly identity safety, representation and adaptive emotion regulation, were more strongly elaborated through the synthesis than in the initial O-ACE formulation. When activities are accessible and acceptable (including online delivery and non-stigmatising framing), young people are more likely to engage, particularly during periods of stress or limited access to professional support. Regular engagement strengthens perceived benefits by providing a consistent avenue for managing feelings. Content features are central in determining whether engagement translates into improved outcomes: emotionally relevant material can support emotional processing, diverse representation and inclusivity can foster identity safety and reduce isolation, and diverse viewpoints and human stories can promote reflection and perspective-taking. Outcomes appear strongest for proximal affective relief (particularly reductions in negative affect) and perceived wellbeing benefits, with more limited and context-dependent evidence for sustained improvements in psychological distress and symptoms of anxiety or depression.

### 3.8 Quality assessment

Quality appraisal was undertaken in accordance with RAMESES standards, with judgements focused on the relevance and rigour of data used to inform programme theory rather than on overall study design or methodological hierarchy (Supplementary Material S4). Across the eleven included studies, all contributed data considered relevant to the development, refinement, or contextual elaboration of context–mechanism–outcome configurations. Studies varied in design and purpose, but most provided sufficiently rich descriptions of context, intervention processes, and participant experiences to support realist explanatory analysis. Qualitative and mixed-methods studies were particularly informative for identifying and elaborating candidate mechanisms, while quantitative and survey-based studies contributed to identifying contextual patterning and outcome regularities across populations and settings. Rigour was assessed at the level of the specific data drawn upon, with data generated through transparent and clearly described methods judged credible for realist use. Conceptual and interpretive papers were retained where they contributed theoretically informative insights, primarily supporting conceptual or contextual elaboration rather than causal testing.

## 4 Discussion

This realist review synthesised evidence on receptive arts and cultural engagement to refine a programme theory explaining how, for whom, and under what conditions such engagement may support anxiety, depression, and psychological distress in young people aged 16–24 years. Across eleven included documents, the overall pattern was clear: receptive engagement appears most reliable for proximal affective outcomes (e.g., reduced negative affect, perceived stress relief, calmness), whereas evidence for sustained symptom-level change is more mixed and conditional. This conclusion is consistent with major syntheses in the arts-and-health field that identify plausible mental health pathways while emphasising heterogeneity and context dependence (1, 2).

A central implication of the refined programme theory is that receptive engagement should be conceptualised as a low-threshold resource that can complement youth mental health systems rather than a standalone intervention expected to generate uniform clinical effects. This complementary role may differ across stages of need with relevance before, alongside and after formal treatment. This aligns with emerging evidence from youth social prescribing pathways and associated best-practice frameworks, which similarly position non-clinical supports as complements to, rather than substitutes for, formal care (50, 51). This framing is particularly relevant given the well-established gap between need and service access in youth populations and persistent barriers to help-seeking, including stigma, confidentiality concerns, and preferences for self-reliance (24). Receptive activities—such as listening to music, visiting museums, watching performances, or engaging with digital cultural content—may be feasible when symptoms reduce motivation, confidence, or social energy, and may be accessed autonomously. This positions receptive engagement as an everyday coping resource and wellbeing support with potential downstream relevance for help-seeking and engagement with care, rather than a wholesale replacement for psychological therapies (1, 2, 51).

The programme theory indicates that one of the most consistent routes to benefit is attentional shift—including absorption, distraction, and momentary psychological distance from stressors. This maps directly onto models of emotion regulation, in which attention deployment can modulate affect in the short-term (52). It is also consistent with evidence that affective symptoms are frequently maintained by repetitive negative thinking (e.g., worry and rumination), and that brief experiences that interrupt these cycles can produce immediate relief even without changing underlying vulnerabilities. Within this review, the strongest controlled experimental support for this proximal outcome pattern came from the “Ways of Being” trial, which demonstrated reduced negative affect over the intervention phase but no sustained effects at follow-up and no differences in psychological distress (46).

However, realist logic requires explicit boundary conditions. Emotion regulation strategies are not uniformly beneficial: meta-analytic evidence suggests that avoidance-linked strategies are more strongly associated with psychopathology, whereas strategies such as reappraisal tend to show more protective associations (53). Applied to receptive engagement, this implies that “escapism” may support recovery when it provides respite and enables re-engagement with daily life, but may be less helpful when it becomes rigid avoidance or reinforces withdrawal. This possibility is consistent with evidence in this review showing moderation by baseline “unhealthy music use” in the Music eScape RCT (42).

Importantly, the mechanisms identified in this review are unlikely to operate uniformly across all young people. Receptive engagement may have limited benefit where content lacks personal relevance, where cultural environments are experienced as exclusionary or psychologically unsafe, or where engagement reinforces maladaptive processes such as rumination, social comparison, distressing memories or emotional avoidance. From a realist perspective, the absence of benefit may therefore reflect different context-mechanism interactions operating for different young people and different forms of receptive engagement, rather than failure of receptive engagement itself. Understanding who benefits, who does not benefit, and under what conditions therefore remains an important priority for future research.

Beyond attentional relief, receptive arts and cultural engagement can plausibly support mental health through reflection and meaning-making, particularly where content provides narrative contact with diverse human experiences. Syntheses of arts and health studies identify meaning, cognition, and emotional processing as plausible mechanisms by which arts engagement may influence wellbeing (1). From a realist perspective, this mechanism may be particularly relevant when young people are navigating developmental transitions and identity formation, and when emotional experiences require integration rather than suppression. This is supported within the included studies with findings suggesting that narrative exposure and storytelling can elicit validation and reappraisal processes, with variation across modality and developmental stage (41).

Although considered together as receptive engagement within this review, different forms of arts and cultural engagement are unlikely to activate identical psychological processes. Music may primarily support affect regulation and physiological calming, literature may encourage perspective-taking and narrative identity, while museums and galleries may facilitate reflection through encounters with artefacts and shared cultural meaning. Nevertheless, these activities share sufficient family resemblance to warrant consideration within a common realist framework because they involve observation, attendance or consumption rather than artistic creation. Future research should examine whether distinct programme theories are required for different forms of receptive engagement.A distinctive contribution of this review is the centrality of belonging and identity safety as mechanism-bearing conditions. Belonging is increasingly understood as a core determinant of wellbeing, and social identity approaches emphasise that feeling connected, recognised and valued can shape mental health through validation, support, and reduced loneliness (54). This is particularly salient for young people who are navigating peer dynamics, social evaluation, and identity development. In the context of receptive engagement, belonging may be produced not only through shared attendance but also through representation and the experience of being “seen” within cultural content, for example through encountering identities, narratives or experiences that feel recognisable. This implies that inclusivity is not a peripheral consideration. Where cultural settings or content are perceived as exclusive, irrelevant, or unsafe, mechanisms linked to validation and connection may not activate. In contrast, identity-affirming cultural environments may reduce isolation and strengthen confidence, particularly for underrepresented groups. This is observed through the included study on identity-affirming cultural spaces (49). Notably, inclusive representation and recognition emerged in the synthesis as more central mechanism-bearing conditions than specified in the initial O-ACE programme theory, illustrating refinement rather than simple confirmation of the original propositions.

The review findings also clarify why more distal symptom change may be harder to detect. The programme theory suggests that intermediate outcomes, such as strengthened emotion regulation, coping confidence, belonging, and identity safety, may act as bridging mechanisms between short-term affective changes and longer-term mental health outcomes. In realist terms, proximal outcomes such as attentional relief or reduced negative affect may arise from a single exposure, whereas broader changes in mental health may depend on repeated activation of mechanisms that generate intermediate outcomes, such as strengthened emotion regulation, increased coping confidence, reduced loneliness, or greater identity safety, which may, under supportive conditions, contribute to longer-term change. This interpretation is consistent with the broader clinical literature distinguishing momentary affect regulation from longer-term regulatory flexibility and coping capacity (52, 53). It also aligns with evidence that adolescence and young adulthood represent a peak developmental window for the onset of mental disorders, during which trajectories may be shaped by sustained exposures and coping patterns rather than isolated experiences (55). Included evidence supports this logic: perceived benefits were stronger among regular users in OAC studies (45) and longer-term improvements were observed over time in both groups in the Music eScape trial, suggesting that repeated practice and routinisation may support the emergence of these intermediate outcomes (42).

### 4.1 Strengths, limitations, and implications

This review's key strength is the realist approach, which supports explanation rather than simple aggregation of effect sizes. By focusing on context–mechanism–outcome configurations and following RAMESES standards, the review provides an actionable account of why receptive engagement may yield reliable proximal benefits while producing more variable distal outcomes (37). However, the evidence base remains small and, although methodologically diverse, a substantial proportion derives from a relatively concentrated cluster of related studies, particularly the O-ACE programme, limiting diversity of contexts and independent replication. To mitigate risks of circularity, O-ACE propositions were treated as candidate mechanisms subject to testing against non O-ACE evidence and were retained, modified or elaborated only where supported across the wider evidence base. While this may strengthen coherence in the emerging programme theory, it also raises the possibility of conceptual reinforcement within a shared programme of work, underscoring the need for independent replication. The predominance of pandemic-era studies also has implications for the transferability of the programme theory. During COVID-19, opportunities for in-person social interaction and cultural participation were substantially restricted, and receptive engagement may have fulfilled needs for connection, distraction and emotional regulation that differ from those in post-pandemic contexts. Likewise, digital engagement became considerably more commonplace during this period. Although the mechanisms identified in this review are likely to remain relevant, their relative importance and the contexts in which they are activated may differ as opportunities for in-person cultural engagement continue to evolve. Future research should examine whether the mechanisms identified in this review operate similarly in post-pandemic contexts and across a wider range of in-person and hybrid cultural experiences. Overall, receptive engagement with arts and culture appears to be a promising, acceptable and scalable route to supporting youth mental health through mechanisms of affective relief, reflection, belonging and identity safety. Its value lies not in substituting for clinical interventions, but in its capacity to operate before, alongside and after clinical care, and in some cases to prevent or reduce the burden on conventional health services. The refined programme theory suggests the most plausible outcomes will be achieved where engagement is accessible, repeated, and inclusive, and where content is relevant and emotionally safe. Future research should test this theory prospectively, explicitly measuring proximal affect alongside longer-term symptom and distress trajectories, and examining moderators including baseline distress, emotion regulation style, loneliness and identity safety. Policy and practice should prioritise equity in design and delivery, ensuring that the benefits of receptive engagement can be realised across diverse youth populations. A particularly fruitful avenue for future research is whether increasing access to receptive arts and cultural engagement for underrepresented young people could function as an accessible gateway, or staged entry point, to more intensive evidence-based interventions.

## Data Availability

Publicly available datasets were analyzed in this study. This data can be found here: Not applicable. No datasets were generated or deposited. All data are derived from published studies cited within the article.
