# Supplementary material for: The role of receptive arts and culture to improve anxiety, depression and psychological distress in young people: a realist review
Source: Front Child Adolesc Psychiatry. 2026 Aug 12;5:1869450. doi: 10.3389/frcha.2026.1869450 (PMC13506279; doi:10.3389/frcha.2026.1869450)
Supplement: Supplementary file 1 [file Table1.docx]

Supplementary Material

S1: Supplementary Table 1: Websites and data archives searched

S2: Search strategy

S3: Supplementary Figure 1: Online Active Community Engagement (O-ACE) programme theory

S4 Supplementary Table 2: Realist quality assessment of included studies (RAMESES-informed)

S5: Candidate context–mechanism–outcome configurations (CMOCs)

S6: Supplementary Figure 2: Initial Programme Theory

**S1: Supplementary Table 1: Websites and data archives searched**

| The Joseph Rowntree foundation | University College London’s Social Biobehavioural Group (UCL-SBB) | The Culture Health and Wellbeing Alliance | The British Library Social Welfare Portal |
| --- | --- | --- | --- |
| Barnardo’s | The Health Foundation | The Wellcome Collection | The Anna Freud Centre |
| Nesta | The King’s Fund | Mind | YoungMinds |
| NCVO | Social Care Online | The National Centre for Creative Health | The World Health Organisation |
| The Centre for Cultural Value | The Mental Health Foundation | The Institute for Volunteering Research | Breathe Arts Health Research |

**S2: Search strategy**

***Concept 1: Youth***

1. child*.ab,kf,ti.
2. teen*.ab,kf,ti.
3. student.ab,kf,ti.
4. young pe*.ab,kf,ti.
5. young adult*.ab,kf,ti.
6. adolescen*.ab,kf,ti.
7. 1 OR 2 OR 3 OR 4 OR 5 OR 6

***Concept 2: Anxiety, depression and distress***
8. anxi*.ab,kf,ti.
9. depress*.ab,kf,ti.
10. distress*.ab,kf,ti.
11. mental health.ab,kf,ti.
12. wellbeing.ab,kf,ti.
13. stress.ab,kf,ti.
14. 8 OR 9 OR 10 OR 11 OR 12 OR 13

***Concept 3: Arts and culture***
15. galler*.ab,kf,ti.
16. theatre.ab,kf,ti.
17. performance*.ab,kf,ti.
18. music.ab,kf,ti.
19. music listening.ab,kf,ti.
20. storytelling.ab,kf,ti.
21. literature.ab,kf,ti.
22. reading.ab,kf,ti.
23. librar*.ab,kf,ti.
24. archive.ab,kf,ti.
25. natural heritage.ab,kf,ti.
26. cultural engagement.ab,kf,ti.
27. cultural participation.ab,kf,ti.
28. digital art*.ab,kf,ti.
29. online cultural engagement.ab,kf,ti.
30. virtual museum*.ab,kf,ti.
31. film.ab,kf,ti.
32. cinema.ab,kf,ti.

33. 15 OR 16 OR 17 OR 18 OR 19 OR 20 OR 21 OR 22 OR 23 OR 24 OR 25 OR 26 OR 27 OR 28 OR 29 OR 30 OR 31 OR 32

34. 7 AND 14 AND 33

**S3: Supplementary Figure 1: Online Active Community Engagement (O-ACE) programme theory**

Supplementary Figure 1: Online Active Community Engagement (O-ACE) programme theory

1 **S4: Supplementary Table 2. Realist quality assessment of included studies (RAMESES-informed)**

| RAMESES quality domain | Bottorff (2015) | Chen et al (2015) | Chmiel et al. (2022) | Goodman & Newman (2014) | Hides et al. (2019) | Reid et al. (2022) | Syed Sheriff et al. (2021) | Syed Sheriff et al. (2021a) | Syed Sheriff et al. (2023) | Syed Sheriff et al. (2023a) | Zhou et al. (2024) |
| --- | --- | --- | --- | --- | --- | --- | --- | --- | --- | --- | --- |
| 1. Research question & purpose | Broad conceptual focus | Clear intervention aim | Explicit aims | Clear intervention aim | Clear intervention focus | Clear evaluation focus | Exploratory aims | Exploratory aims | Descriptive / evaluative | Descriptive / evaluative | Clear evaluative aim |
| 2. Relevance to programme theory | Conceptual only | Moderate–High (mechanism informing) | Moderate (contextual) | High (mechanism-rich) | High | Moderate | High | High | High | High | Moderate (context–outcome; some mechanism informing) |
| 3. Appropriateness of design | Interpretive synthesis | Randomised controlled trial | Cross-sectional survey | Quasi-experimental | Trial / feasibility | Process / outcome eval | Qualitative | Qualitative | Mixed / qualitative | Mixed / qualitative | Pre–post survey evaluation |
| 4. Context description | Cultural context | Moderate (student setting; intervention context clear) | Strong macro-context | Limited (school-based) | Detailed youth service context | Partial | Rich service context | Rich service context | Rich implementation context | Rich implementation context | Strong contextual description |
| 5. Transparency of methods | Narrative | Clear | High | Clear | Clear | Clear | Partial | Partial | Partial | Partial | High |
| 6a. Relevance (RAMESES §6) | Theory-informing | CMOC-informing | Context–outcome | CMOC-informing | CMOC-informing | Partial | CMOC-informing | CMOC-informing | CMOC-informing | CMOC-informing | Context–outcome / partial CMOC-informing |
| 6b. Rigour (RAMESES §6) | Anecdotal | Validated tools; physiological + self-report outcomes | Large sample | Validated tools | Robust methods | Robust methods | Credible qual data | Credible qual data | Credible mixed data | Credible mixed data | Large sample; validated affect measures |
| 7. Unintended outcomes | Not addressed | Limited | Limited | Not addressed | Limited | Limited | Some discussion | Some discussion | Some discussion | Some discussion | Limited |
| 8. Reflexivity | Authorial stance | Minimal | Partial | Minimal | Partial | Partial | Some reflexivity | Some reflexivity | Some reflexivity | Some reflexivity | Partial |
| 9. Contribution to theory refinement | Conceptual scaffolding | Moderate-Strong | Contextual calibration | Strong | Strong | Moderate | Strong | Strong | Strong | Strong | Moderate |
| Overall realist judgement | Data were used to inform CMOC refinement | Data were used to inform CMOC development | Data were used to inform CMOC refinement | Data were used to inform CMOC development | Data were used to inform CMOC development | Data were used to inform CMOC refinement | Data were used to inform CMOC development | Data were used to inform CMOC development | Data were used to inform CMOC development | Data were used to inform CMOC development | Data were used to inform CMOC refinement |

**Footnote:** In accordance with the RAMESES quality and reporting standards, judgements of quality were made at the level of the data contributing to theory development rather than at the level of the study as a whole. Evidence was appraised for its relevance (i.e. whether it could contribute to the development, refinement, or testing of context–mechanism–outcome configurations and programme theory) and for its rigour (i.e. whether the methods used to generate the specific data drawn upon were credible and trustworthy). Studies were not excluded solely on the basis of design or traditional hierarchies of evidence, but were included where they contributed substantively to realist explanatory analysis.

**S5: Candidate context–mechanism–outcome configurations (CMOCs)**

1. When receptive arts activities are free or low cost (C), young people may be more likely to engage (O), because low practical barriers increase perceived feasibility and autonomy (M).
2. When receptive arts activities are available online and on demand (C), young people may be more likely to engage (O), because flexibility and immediacy support access and control (M).
3. When online engagement environments are perceived as unsafe or unmoderated (C), engagement may be avoided or benefits weakened (O), because psychological safety needed for positive engagement is undermined (M).
4. When receptive arts activities are framed as non-clinical and open to all (C), young people may be more willing to engage (O), because stigma-related barriers are reduced (M).
5. When young people feel overwhelmed or circumstances feel beyond their control (C), receptive engagement may reduce distress or negative affect (O), because attentional shift and temporary psychological distance provide relief (M).
6. When young people feel isolated (C), receptive engagement may reduce isolation and improve wellbeing (O), because human connection, affiliation or perceived belonging are strengthened (M).
7. When young people experience societal or developmental pressures (C), receptive engagement may reduce stress (O), because attentional focus is redirected away from stressors (M).
8. When young people encounter narratives showing others navigating similar challenges (C), receptive engagement may improve coping (O), because reflection and cognitive reappraisal are activated (M).
9. When young people seek emotionally safer alternatives to emotionally draining digital environments (C), receptive engagement may reduce anxiety (O), because psychologically safer inputs provide emotional containment (M).
10. When young people experience stigma or marginalisation (C), receptive engagement may support wellbeing (O), because identity exploration and validation are enabled (M).
11. When young people are processing difficult past experiences (C), receptive engagement may support coping (O), because symbolic or narrative content enables meaning-making (M).
12. When a range of cultural activities is available (C), young people may be more likely to find something engaging (O), because choice increases relevance and personal fit (M).
13. When content aligns with emotional needs or preferences (C), receptive engagement may support mood regulation and coping (O), because young people can use content adaptively for emotion regulation (M).
14. When cultural content reflects identities and experiences that feel recognisable and affirming (C), engagement may strengthen belonging and confidence (O), because young people feel seen and validated (M).
15. When content exposes young people to diverse perspectives and human stories (C), receptive engagement may improve coping and reduce distress (O), because perspective-taking and empathy are activated (M).
16. When engagement is regular and routinised (C), benefits may be more likely to persist (O), because engagement functions as an ongoing self-management resource (M).
17. When engagement connects young people to groups or communities organised around shared cultural interests (C), mental health outcomes may improve (O), because shared identity and belonging are strengthened (M).
18. When receptive engagement is experienced as voluntary and self-directed (C), acceptability and sustained engagement may increase (O), because autonomy and agency are supported (M).

**S6: Supplementary Figure 2: Initial Programme Theory**

Supplementary Figure 2: Initial Programme Theory
